# A Novel Remote Rehabilitation System with the Fusion of Noninvasive Wearable Device and Motion Sensing for Pulmonary Patients

**DOI:** 10.1155/2017/5823740

**Published:** 2017-05-03

**Authors:** Chuang-Kit Tey, Jinyoung An, Wan-Young Chung

**Affiliations:** Department of Electronic Engineering, Pukyong National University, Busan 608-737, Republic of Korea

## Abstract

Chronic obstructive pulmonary disease is a type of lung disease caused by chronically poor airflow that makes breathing difficult. As a chronic illness, it typically worsens over time. Therefore, pulmonary rehabilitation exercises and patient management for extensive periods of time are required. This paper presents a remote rehabilitation system for a multimodal sensors-based application for patients who have chronic breathing difficulties. The process involves the fusion of sensory data—captured motion data by stereo-camera and photoplethysmogram signal by a wearable PPG sensor—that are the input variables of a detection and evaluation framework. In addition, we incorporated a set of rehabilitation exercises specific for pulmonary patients into the system by fusing sensory data. Simultaneously, the system also features medical functions that accommodate the needs of medical professionals and those which ease the use of the application for patients, including exercises for tracking progress, patient performance, exercise assignments, and exercise guidance. Finally, the results indicate the accurate determination of pulmonary exercises from the fusion of sensory data. This remote rehabilitation system provides a comfortable and cost-effective option in the healthcare rehabilitation system.

## 1. Introduction

Chronic respiratory diseases are diseases of the respiratory tract and structures of the lung. Some of the most common are asthma, chronic obstructive pulmonary disease (COPD), and occupational lung diseases. COPD is the most severe among the chronic respiratory diseases. One of the main causes of chronic respiratory diseases across high-, middle-, and low-income countries is tobacco smoking. According to World Health Organization (WHO) estimates, more than 3 million people died from COPD in 2012, which corresponds to 6% of all deaths globally [1]. The number is still growing, and research has shown that COPD will become the third leading cause of death worldwide by 2020 [2]. In high-income countries such as the United States, mortality rates for COPD doubled between 1970 and 2002 [3].

The importance of rehabilitation for pulmonary patients has been recognized by related standard and document, for example, ATS/ERS (American Thoracic Society/European Respiratory Society) standards [4] and the Global Initiative for Chronic Obstructive Lung Disease (GOLD) document [5]. Complete recovery is generally difficult for patients who suffer from chronic respiratory diseases; however, these diseases can be treated. Therefore, it is recommended that patients undergo pulmonary rehabilitation (PR), which helps them sustain the functionality of their lungs throughout their lifetimes and not aggravate their illness by misusing their lungs [6]. In addition, patients usually follow respiratory exercises to improve lung functionality. As a result, PR has been recognized as a treatment that enhances functional ability and improves dyspnea and better quality of life [7].

To regain functionality or to cope with the loss of functionality, intensive rehabilitation progression is required. Various types of rehabilitations for indoor and outdoor training can be adopted. In the indoor exercise for pulmonary rehabilitation, aerobic exercise (walking, cycling, and treadmill), strength training (upper extremity and lower extremity), breathing exercises, self-management, inspiratory muscle training, and home exercise were introduced for effective pulmonary rehabilitation [8]. According to [9, 10], training at least three times per week is necessary, and patients should attend regular supervision of rehabilitation sessions to obtain optimal exercise benefits. Thus, frequent attendance at rehabilitation sessions is the optimal solution, where the patient is supervised by medical professionals throughout the session.

However, healthcare costs are constantly increasing owing to the high demand for medical care, and this leads to a shortage of medical professionals and medical infrastructures. Owing to the high costs, not all patients can attend sessions frequently. Thus, many patients choose to continue performing rehabilitation exercises at home. Unfortunately, however, performing rehabilitation exercises at home is less effective because of the lack of supervision or reduced levels of motivation [11]. In addition, multiple biomedical devices attached to the patient's body cause discomfort in operating the biomedical device. These issues have raised community concerns regarding pulmonary healthcare systems. Telemedicine used in the remote delivery of healthcare via information and communications technology is one of the promising approaches to decrease healthcare expenses and still achieve similar outcomes.

Numerous approaches have been proposed of healthcare system for pulmonary disease. Methods have been developed based on a single signal such pulse oximetry [12], several biomedical sensors for monitoring [13], and mobile-health system to manage patients at home [14], and wearable platform contains of multiple biomedical sensor to monitoring health level [15]. Above methods provide good accuracy for monitoring health condition and alert patient to prevent aggravate symptoms. However, above approaches are missing necessary component to control and relieve symptoms and prolong functional capacity.

In this study, we propose a novel system which uses data fusion of motion-sensing data and biomedical data of a patient for remote pulmonary rehabilitation exercise system. A motion-sensing stereo-camera system and a wearable biomedical device are combined, and the sensor fusion of motion-sensing data and biomedical signal provides optimum effect of remote pulmonary rehabilitation. The wearable biomedical device is embedded with a photoplethysmogram (PPG) sensor to collect vital signs not only for postanalysis but also as parameters along with skeletal-tracking data to perform the rehabilitation exercise. The system detects a patient's posture and movement and provides automated feedback if required. This concept can improve the rehabilitation system such that the dependence on the supervision decreases and self-management ability increases by a rear time feedback. Meanwhile, exercise repetitions and health status data are uploaded to a web-based server–client computing system. By using such a web server database and web application, both patients and medical professionals can seamlessly access the medical record when required.

## 2. System Design and Implementation

**Figure 1:**
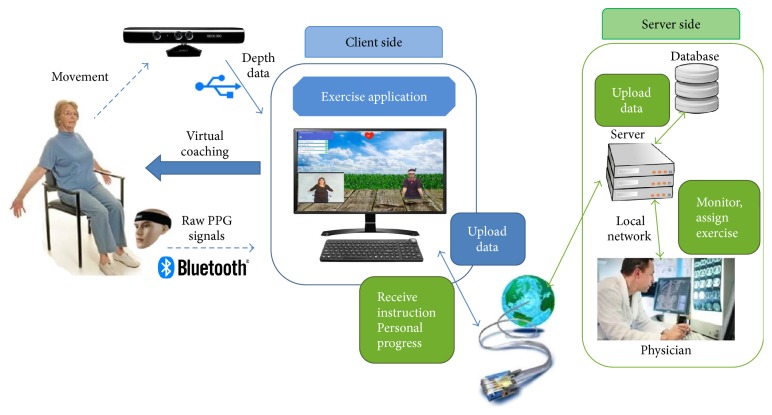
Overall system architecture of the proposed system.

The fusion wearable device and motion-sensing exercise-monitoring system for PR was intended to measure biomedical data such as heart rate and breathing rate while the patient follows coaching videos for performing physical activity. This provides new possibilities, wherein the patient is always under supervision while accomplishing physical activity, or the professional clinician can regularly monitor patient progress and analyze patient health condition using the biomedical data generated during the exercise. It is crucial for patients to see the progress of their rehabilitation program at any time and at any location; the health data are effortlessly updated onto a web server for professional health monitoring and further analysis.  Figure 1 shows the overall system architecture of the proposed system, the fusion wearable device, and the motion-sensing exercise-monitoring system. The proposed system is further classified into four segments: biomedical signal analysis, physical exercise gestures recognition, natural user interface, and rehabilitation application. Biomedical signal analysis is intended for heart rate and respiration rate monitoring. Physical exercise gestures recognition defines relevant body parts and uses skeletal data and biomedical data as parameters for exercise recognition. Finally, an online application and user interface is developed to display results and to instruct the patient via the web server and to synchronize health data for monitoring.

### 2.1. Biomedical Signal Analysis

**Figure 2:**
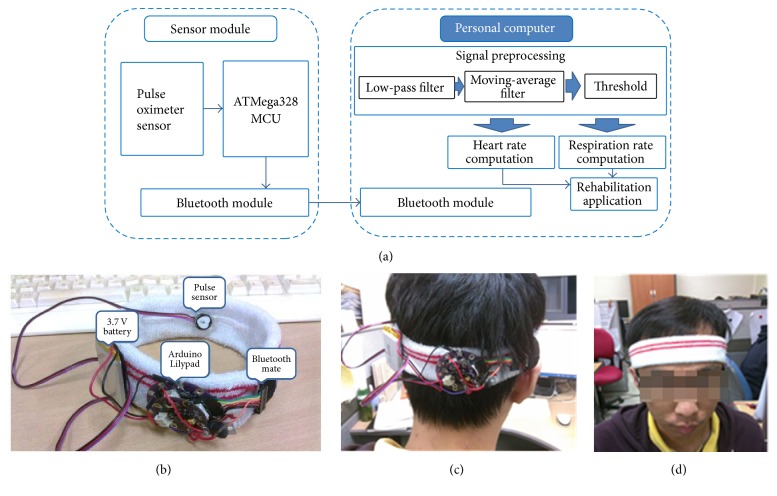
Proposed wearable biomedical device: (a) block diagram of device system architecture; (b) wearable biomedical device; (c) back view; (d) front view.

Figure 2 illustrates the proposed wearable biomedical device and a block diagram of system architecture is observed in Figure 2(a). The system consists of two major components: (i) a sensor module built using an ATmega microprocessor, a Bluetooth module, and a pulse oximetry sensor (SEN-11574, Sparkfun Electronics, USA), and (ii) a personal computer (PC) that receives and processes the signals as an input for the rehabilitation application. Here, the adopted pulse sensor clips onto hairband to obtain the PPG signal from patient (Figure 2(b)) and the clear PPG pulse is observed without error.

A wearable biomedical device as shown in Figures 2(b)–2(d) is embedded into a reflection-type pulse oximeter sensor with a sampling frequency of 50 Hz and a Bluetooth module for wireless transmission to the receiver PC (Arduino microcontroller and Bluetooth Mate). The application on the PC processes the raw PPG signal into usable information. First, the common information from the PPG signal is processed into user heart rate per minute by computing the peak-to-peak interval. Subsequently, the respiration rate can be determined from a raw PPG signal using a PPG-derived respiration method. Noise cancellation is crucial at this stage in order to remove power noise and motion artifacts from the raw PPG signals and to reduce the effect of noise that influences later-stage decisions.

Initially, the PC receives the raw PPG signal from the sensor module, and this signal goes through a preprocessing process for noise reduction filtering and peak detection. In the preprocessing algorithm, (i) a low-pass filter with a cutoff frequency of 4 Hz is applied for high analog signal noise filtering.

Next, (ii) a moving-average filter is applied to the filtered signal to reduce random noise while preserving sharp step responses. The moving-average filter can be created using (1). In the equation, *x*[ ] and *y*[ ] denote input and output signals, respectively, and parameter *M* represents the filter length.

(1) $$\begin{array}{c} y\left[\;i\;\right]=\overset{\left(M-1\right)/2}{\underset{j=-\left(M-1\right)/2}{\sum }}x\left[\;i+j\;\right]. \end{array}$$

Next, (iii) a noise-free PPG signal is obtained, and signal analysis can be performed to evaluate the health condition of a user. A stipulated threshold is used to identify the peak of the signals based on the point of steepest slope to determine the extreme value in the signal. Finally, peaks and valleys are detected after thresholding, and the values are used for computing heart rate and respiration rate.

Respiratory-induced intensity variation (RIIV) is present in the PPG signal baseline [16]. The RIIV is considered to be triggered by skin-blood arterial fluctuations stimulated by the respiratory variations in intrathoracic pressure transmitted to the measurement device by the venous system [17]. The heart and respiration rates can be monitored by obtaining the RIIV and the cardiac synchronous component from the PPG signal. Various signal processing tools such as time-frequency analysis using wavelets and short-time Fourier transform and time series analysis using both parametric and nonparametric modeling techniques have been utilized to prove PPG as consistent measurement parameter of respiratory rate along with heart rate [18].

### 2.2. Exercise Gesture Recognition

For exercise gesture recognition, the appropriate body parts are identified to perform selected exercises, and then, the entire exercise is divided into segments of certain durations. Each segment illustrates the movement of each related body part in terms of horizontal, vertical, depth translation, and angle between joints. A state machine was developed for recognizing PR exercises in real time using a motion-sensing camera and a wearable biomedical device. The state machine model (SMM) algorithm is incorporated into this system. At the beginning of the SMM, once all conditions have been met for one particular state, state transition occurs. The user is required to go through each state in progression from start to end for an exercise gesture to be recognized as completed in a cycle.

### 2.3. Rehabilitation Application

The user interface of the exercise application displays information such as daily repetitions of the exercise, instructions for performing selected exercises, and the coaching video that provides guidelines for the selected exercises. In the main menu of the application, the user can navigate the user interface using hand movements, for example, moving the hand forward to select, grab, and swipe to move left and right. The exercise application is connected to a web server that receives instructions given physically and updates data such as heart rate and completion of a selected exercise for further evaluation.

## 3. Exercise Clarification and Details for the Proposed System

### 3.1. Exercise Clarification

In actual pulmonary rehabilitation, various environment factors (ventilatory limitation, gas exchange limitation, etc.) and patient body condition (body composition, cardiac dysfunction, and respiratory muscle dysfunction) should be considered [19]. With considered factors, the required exercise training and suitable training schedule are quite different. Several exercises, such as aerobic exercise (walking, cycling, and treadmill), strength training (upper extremity and lower extremity), breathing exercises, self-management, nutritional support, inspiratory muscle training, and home exercise prescription and education can be adopted for pulmonary rehabilitation [8]. Among various exercise training, pulsed lib breathing with open-chest exercise is adopted and it is applied to the proposed rehabilitation system for demo-exercise. This exercise is related to pulmonary gas exchange and respiratory muscle, where efficient pulmonary gas exchange and robust respiratory muscle are very important to improve functional capacity and exercise endurance [8, 20]. The novel concept system is considered for home-based rehabilitation and further details are discussed in next section.

### 3.2. Details for the Proposed System

To obtain optimal exercise benefits, training at least three times per week is necessary, and patients should attend regular supervision of rehabilitation sessions [9, 10], where supervision and feedback from physician supervisor are significant. Meanwhile, home-based exercise for pulmonary rehabilitation, which offers the greatest convenience for the patient, was introduced and showed similar physiologic benefit to that of hospital-based one [20, 21]. The adopted concept exercise, which is the pulsed lib breathing with open chest, is for home-based demo-exercise and the rehabilitation process consists of three parts as follows:

- Starting pose: Subjects sit in a chair with back support. Hip and knee joints are at a 90-degree angle, and feet are kept flat on the floor.
- Inhalation: Spread out objects' arms back and keep chest forward and simultaneously take a deep breath.
- Exhalation: Straighten object's back and make arms forward with palm to palm and simultaneously breathe out deeply with pulsed lip.

Here, the process is performed with reference video on the screen. Objects movement is recognized by motion-sensing camera system and program moves to next step when objects' gestures are correct. Therefore, patients can check their problems on motion without the supervision and get feedback of each progress in real time. Furthermore, each process is automatically saved and uploaded on web server for further analysis and prescription at any time. After that, the physician supervisor can monitor and analyze the health status of the patient from a remote site either in real time or at a later time. Referring to similar home-based exercise [20], five times a week for 8 weeks is required to achieve exercise benefits. The adopted exercise also requires training at least five times a week for 8 weeks and training with increase of dumbbell weight (0.5 lb to 4 lb) helps improve respiratory muscle [20].

## 4. Results and Discussion

The evaluations of PPG-derived respiratory rate are conducted using a MATLAB tool set. A PPG database (MIMIC II Waveform Database) evaluates the effectiveness of detecting a respiratory rate from a PPG signal. The rehabilitation application is developed using C# and Windows Presentation Foundation.

### 4.1. Biomedical Signal Analysis

#### 4.1.1. Heart Rate Computation

The heart rate can be determined by detecting the systolic peak of the PPG signal. Subsequently, the time difference between successive systolic peaks is referred to as the peak-to-peak interval. The heart rate can be computed as

(2) $$\begin{array}{c} Heart\;\;rate\;\left(\;bpm\;\right)=\frac{60}{Peak-Peak\;\;interval\;\left(s\right)}. \end{array}$$

#### 4.1.2. Respiration Rate Computation

**Figure 3:**
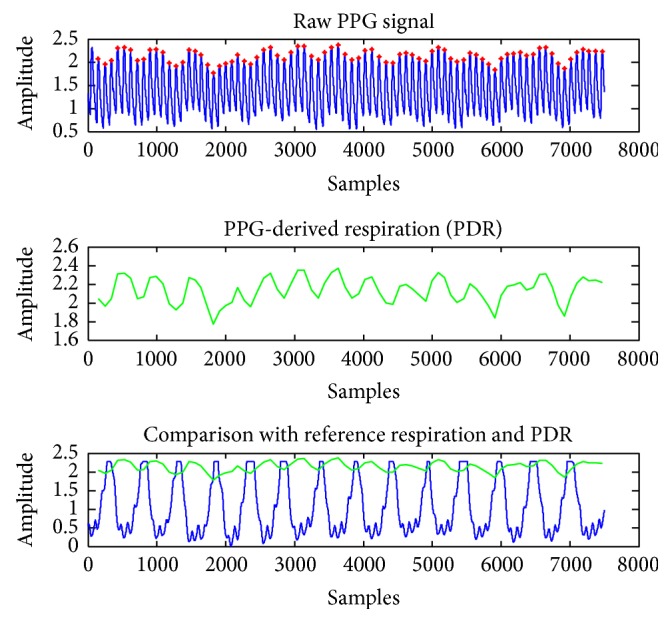
Comparison between the PDR signal and the reference respiration signal.

Several techniques can be applied to extract PPG-derived respiration (PDR) data. The peak detection value extracts respiratory-induced intensity variations from the PPG signal baseline. With the intensity variations, the pattern of inhalation and exhalation, which is directly related to respiratory rate, can be observed. An overview of respiratory patterns from the PPG signal is presented in Figure 3, where first subplot shows the sampled PPG signal, second subplot shows peak detection of the PPG pulse (red points at first subplot), and reference respiration signal is observed at final subplot. During inhalation, the peak value decreases and the valley value increases. Further, it decreases the distance between the peak and the valley parameter. The reverse effects are seen during exhalation. The pattern of the measured PDR is exactly the same as that of the reference respiration signal as shown in Figure 3 (final subplot). In our proposed system, PPG signals are measured in addition to heart rate and PDR, which are computed in real time. Further, the biomedical data are used as parameters in the exercise gesture recognition module.

### 4.2. Exercise Gesture Recognition

**Figure 4:**
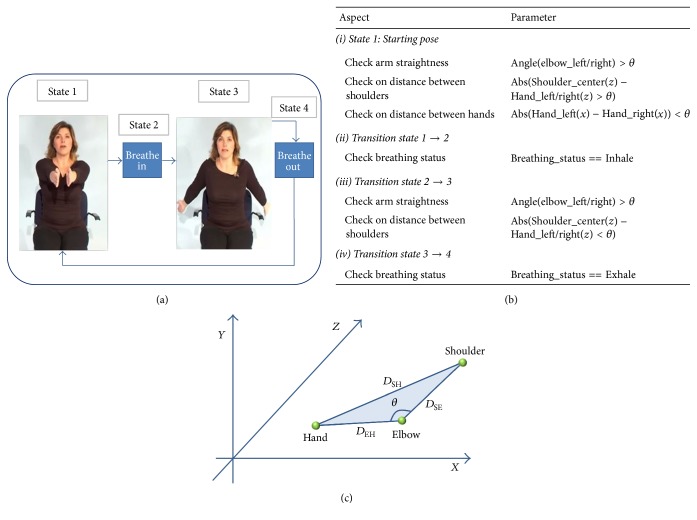
Parameterization of target exercises: (a) SMM for open chest; (b) parameters of open-chest exercise; (c) computed angle between elbow joints.

Patient's movement is captured with motion-sensing camera SDK that provides 3D skeletal joint tracking. The 3D coordinates are obtained from skeletal joints tracking to perform angle measurement computation for detection and evaluation of the target exercises chosen (open chest, snow angel, lat pulldown, etc.). Figures 4(a) and 4(b) illustrate SMM structure, for building an evaluation algorithm. Every parameter can be clearly programmed into the system. Followed by stating the exercise threshold range *θ*, each of the states can be evaluated.  Figure 4(c) shows the calculation for the angle of elbows joint measured in degrees *θ*, which is used as one of the parameters in Figure 4(b). The 3D coordinates of the shoulder, elbow, and hand joints are used to compute the Euclidean distance as shown in (3), and by applying the law of cosine, as in (4), the angle of the joint is determined. The parameters can be changed according to the patient's condition and progress by physician, in which, it offers a personalized set of exercises that best suit for that particular patient.

(3) D=x1−x22+y1−y22+z1−z22

(4) θ=arccos⁡DSE2+DEH2−DSH22DSE2·DEH2.

### 4.3. Proposed System

**Figure 5:**
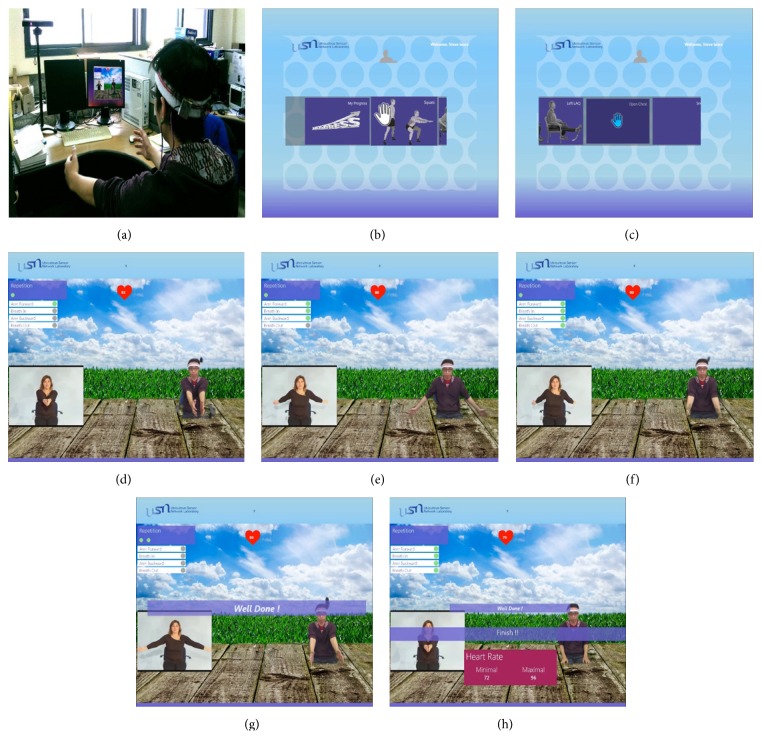
Demonstration of the proposed system: (a) user following guidelines provided by the system in real time; (b) main menu; (c) user selects exercise; (d) state one of open-chest exercise; (e) state three of open-chest exercise; (f) state four of open exercises; (g) completion of a repetition of an exercise; (h) completed set of exercises and heart rate information.

The proposed system describes a PR system to guide and improve the health status of patients with chronic respiratory diseases and to create a home-based PR system that is more applicable and cost-effective.  Figure 5(a) shows a user following the guideline provided by the system in real time. The headband of the user is wearable biomedical device in Figure 2(a).  Figure 5(b) shows the main menu page for the rehabilitation application; the user can use hand tracking to navigate and select items from the menu as shown in Figure 5(c). This system is composed of a motion-sensing camera, a wearable PPG device placed on the forehead, and a PC-based rehabilitation application. Human tracking using the motion-sensing feature's analysis system provided by Microsoft enables the motion-sensing camera to recognize people and track their movements. The raw PPG data are transmitted to a PC via Bluetooth and used to evaluate their health status based on biomedical signals such as heart and breathing rates. The system can also measure breathing data, which is set as a parameter along with skeletal data for an exercise recognition algorithm. The user interface of the system, as illustrated in Figures 5(d)–5(h), provides guidelines to help users perform exercises as accurately as intended.

## 5. Conclusion

This study proposed a rehabilitation system that combines a wearable PPG device and a motion-sensing camera for support and evaluation of PR exercises. PDR and physical exercise gestures are detected seamlessly in real time. Patient-generated data such as heart rate and exercise record from throughout the rehabilitation exercise are automatically logged and synchronized to the healthcare management server where a medical professional can monitor and analyze the health status of the patient from a remote site either in real time or at a later time. The proposed remote rehabilitation system receives personalized prescription exercises from medical professionals through the Internet, simultaneously using a motion-sensing rehabilitation application and a biomedical sensing device to provide instructive experience for the patient when the patient follows the guidance for performing the exercises. Moreover, the system represents a comfortable and cost-effective solution for patients. The system can be easily set up in any given place with an Internet connection, and it reduces the frequency of patient visits to a medical center. In addition, the medical professional can still keep track of patient condition and progress without any extra expenses.

## 6. Future Perspective

Considering several standards and document for COPD, such as ATS/ERS (American Thoracic Society/European Respiratory Society) standards [4] and the Global Initiative for Chronic Obstructive Lung Disease (GOLD) document [5], pulmonary rehabilitation has been popularized and an importance of management of patients with COPD has become recognized in a relatively short time. Various pulmonary rehabilitations have been developed along several lines. Although there are several rehabilitation exercises to alleviate COPD, it is not easy that all patients attend the effective pulmonary rehabilitation programs, where many of pulmonary patients are old, weak, or poor or have additional physical defects. Therefore, the flexible rehabilitation should be developed to cover various perspectives as follows.

First, pulmonary rehabilitation should be made available to all patients who have physical defects or who are in a specific location or circumstance. Additionally, more research is required to minimize financial burdens with long-term medical care. Furthermore, an alternative to alleviating a shortage of professionals and medical infrastructures is needed. For instance, home-based rehabilitation can be a good alternative, which can cover various challenges above, under assumption that patients are sufficiently educated. In this point of view, we expect that more home-based remote pulmonary rehabilitation (PR) schemes could be invented and efforts for the cost-reduction of PR could be made in the next few years. The considered remote PR system is a good example for convenient and cost-effective solution. The proposed multimodal sensors-based application could be one of the significant materials for future studies about a remote PR system.
